# *In vitro* Evaluation of Novel Sustained Release Microspheres of Glipizide Prepared by the Emulsion Solvent Diffusion-Evaporation Method

**DOI:** 10.4103/0975-1483.62210

**Published:** 2010

**Authors:** P Phutane, S Shidhaye, V Lotlikar, A Ghule, S Sutar, V Kadam

**Affiliations:** *Department of Pharmaceutics, Bharati Vidyapeeth’s College of Pharmacy, Sector 8, C.B.D. Belapur, Navi Mumbai - 400 614, India*; 1 *Vivekanand Education Society’s College of Pharmacy, Behind Collector Colony, Chembur (E), Mumbai - 400 074, Maharashtra, India*

**Keywords:** Microspheres, Glipizide, ethyl cellulose, Eudragit^®^ S100, emulsion solvent diffusion-evaporation technique, sustained release

## Abstract

The objective of the current investigation is to reduce dosing frequency and improve patient compliance by designing and systematically evaluating sustained release microspheres of Glipizide. An anti-diabetic drug, Glipizide, is delivered through the microparticulate system using ethyl cellulose as the controlled release polymer. Microspheres were developed by the emulsion solvent diffusion-evaporation technique by using the modified ethanol,-dichloromethane co-solvent system. The polymer mixture of ethyl cellulose and Eudragit^®^ S100 was used in different ratios (1:0, 1:1, 2:3, 1:4 and 0:1) to formulate batches F1 to F5. The resulting microspheres were evaluated for particle size, densities, flow properties, morphology, recovery yield, drug content, and *in vitro* drug release behavior. The formulated microspheres were discrete, spherical with relatively smooth surface, and with good flow properties. Among different formulations, the fabricated microspheres of batch F3 had shown the optimum percent drug encapsulation of microspheres and the sustained release of the Glipizide for about 12 h. Release pattern of Glipizide from microspheres of batch F3 followed Korsmeyers-peppas model and zero-order release kinetic model. The value of ‘n’ was found to be 0.960, which indicates that the drug release was followed by anomalous (non-fickian) diffusion. The data obtained thus suggest that a microparticulate system can be successfully designed for sustained delivery of Glipizide and to improve dosage form characteristics for easy formulation.

## INTRODUCTION

The limitations of the most obvious and trusted drug delivery techniques, such as conventional drug delivery system (DDS), have been recognized for some time now, the most important limitation of them being the patient incompliance due to frequent medication. This limitation can be overcome by modifying existing DDS. An appropriately designed sustained release (SR) or controlled release DDS can be a major step toward solving the problem associated with conventional DDS.[1,2] The SR DDS also have solutions for other limitations of the conventional DDS such as undesirable side effects due to fluctuating plasma drug level, inability to maintain adequate drug concentration in plasma for therapeutic effect, larger doses than those required by the cells have to be administered in order to achieve the therapeutic concentration, causing the undesirable, toxicological and immunological effects in non-target tissues.

Some drugs are readily absorbed from the GI tract, but easily eliminated from the body via excretion on account of its short half-life, requiring concomitant drug administration. Formulating an oral controlled release dosage form for these classes of drugs can be most beneficial as they release drug slowly in GIT and maintain constant drug levels in plasma for the extended period.[3] SR dosage forms, based on multiparticulate systems have attracted much attention due to their several benefits in reducing risk of dose dumping, and local irritation as the individual units can pass randomly through the pylorus and distribute widely in the GI tract[4] producing more predictable drug release profiles.

Glipizide is one of the most rapid and short acting second-generation blood-glucose-lowering drug belonging to class of sulphonylurea[5] and specially used in type II diabetes (non-insulin-dependent diabetes mellitus). The recommended dose range is 2.5-20 mg daily.[5] The absolute bioavailability is close to 1, thus it belongs to Biopharmaceutical Classification System (BCS) Class 2.[6] Gastrointestinal absorption of Glipizide is uniform, rapid, and essentially complete with relatively short elimination half life (3.4 ± 0.7 h).[7] The development of controlled release dosage forms thus, would clearly be advantageous. The characteristics of the drug such as short half life, low dose, and therapeutic use in chronic disease make it a suitable candidate for sustained release formulation.

The objective of the present invention is to develop and evaluate a sustained release microparticulate system of Glipizide in order to extend the drug release for about 12 h of duration and. The microspheres were evaluated for particle size, densities, flow properties, morphology, recovery yield, drug content, and *in vitro* drug release behavior.

## MATERIALS AND METHODS

### Materials

The active ingredient Glipizide was obtained as gift sample from Cipla Pharmaceuticals, Mumbai. Eudragit^®^ S100 was procured from Evonik Degussa India Pvt. Ltd., Mumbai and Ethyl cellulose from Central Drug House (P) Ltd., New Delhi. Polyvinyl alcohol (PVA) was obtained from Research Lab., Mumbai. Ethanol, n-butanol, and dichloromethane used were of analytical grade purchased from S.D. Fine chemicals Limited, Mumbai, India. Double distilled water was used throughout the study.

### Methods

#### Formulation of sustained release Glipizide microspheres

Before initiating formulation of microspheres, compatibility of Glipizide with different excipients was studied using the techniques like compatibility test for solid dosage form on lab scale[8] and DSC testing. Excipients used in formulation batches were found to be compatible with Glipizide.

Formulation of drug-loaded microspheres was carried out by the emulsion solvent diffusion-evaporation method. The polymers ethyl cellulose and Eudragit^®^ S100 were used in different ratios with formulation batches F1 to F5, these ratios were shown in Table 1. The preferred ratio of 1:19 of Glipizide to polymer was used for all batches. Initially a solvent mixture of ethanol: dichloromethane: n-butanol was prepared in the ratio of 8:5:2 considering their volumes. An accurately weighed quantity of Glipizide (50 mg) and enteric polymer Eudragit^®^ S100 along with ethyl cellulose was co-dissolved at room temperature in a solvent mixture. This solution was introduced into 1000 ml of 0.4% PVA aqueous solution at room temperature and dispersed to form emulsion at stirring rates of 200 rpm using a mechanical stirrer equipped with 4-blade propeller. Agitation provided by stirrer breaks the poured polymer solution to form an oil-in-water (O/W) type emulsion. This emulsion was then stirred for about 20 min at room temperature. After stirring, the solidified microspheres were recovered by filtration, washed with phosphate buffer (pH 7.4 ± 1) to remove all non-encapsulated drug, and further with distilled water to wash off PVA solution. Recovered microspheres were dried at 50°C for 12 h to remove solvents.

**Table 1:** Formulae of Glipizide microspheres with variable polymer ratios

Formulation batches*#	Ratio of ethyl cellulose to Eudragit^®^ S100	Quantity of polymer used (mg)		Quantity of Glipizide (mg)
		Ethyl cellulose	Eudragit^®^ S100	
F1	1:0	950	0	50
F2	1:1	475	475	50
F3	2:3	380	570	50
F4	1:4	190	760	50
F5	0:1	0	950	50

* Stirring carried out at room temperature;

# Ratio of solvent used in each formulation was 8:5:2 (Ethanol:DCM:n-butanol)

### Evaluation of microspheres

#### Micromeritic properties

Microspheres were characterized for their micromeritic properties such as particle size, shape, bulk density, tapped density, compressibility index, Hausner’s ratio, and angle of repose. The size was measured using an optical microscope with the help of a calibrated ocular and stage micrometer, and the particle size range was obtained by measuring size of about 100 particles.[9]

Densities were derived as follows: An exact quantity ‘M’ of microsphere was taken and was placed into a measuring cylinder. Volume ‘V’ occupied by the microspheres was noted without disturbing the cylinder and bulk density was calculated using the following equation;[9]

$$\mathrm{Bulk}\;\mathrm{density}\left(P_{b}\right)\;=\;\frac{M}{V}$$

The tapping method was used to determine the tapped density in which the cylinder containing known amount (M) of microspheres was subjected to a fixed number of taps (approximately 100) until the bed of microspheres had reached the minimum. The final volume after tapping ‘V_o_’ was recorded and the tap density was calculated by the following equation:

$$\mathrm{Tapped}\;\mathrm{density}\left(P_{p}\right)={\frac{M}{V}}_{0}$$

Angle of repose, Hausner ratio, and Carr index (% compressibility index) were determined to predict flowability. A higher Hausner ratio indicates greater cohesion between particles, while a high Carr index is indicative of the tendency to form bridges. Angle of repose[9] of the microspheres, is the maximum angle possible between the surface of the pile of microspheres and the horizontal plane, was obtained by fixed funnel method using the formula;

$$\mathrm{Angle}\;\mathrm{of}\;\mathrm{repose}\left(\theta \right)=\tan^{-1}\left(\frac{2h}{d}\right)$$

Where, h is height and d is the diameter of the microsphere pile that is on a paper after making the microspheres flow from the glass funnel.

Hausner ration and Carr index were calculated using the formulae:

$$\begin{array}{l} \mathrm{Carr}\;\mathrm{index}\;\mathrm{or}\;\%\mathrm{compressibility}\;\mathrm{index}\;\mathrm{or}\;C=\left[1–\frac{V_{o}}{V}\right]\;\times 100\\ \mathrm{Hausner}\;\mathrm{ratio}=\frac{100}{100+C} \end{array}$$

Here, V and V_o_ are the volumes of the sample before and after the standard tapping, respectively and C is Carr index.

#### Morphology

The surface topography, particle size, morphology, and internal cross-sectional structure of the microspheres were explored by using the technique like scanning electron microscopy (SEM).[10] The ultra-structural features were analyzed by JEOL Scanning Electron Microscope (JSM-5400). Before the samples were analyzed, dry microspheres were placed on an electron microscope brass stub and coated with gold in an ion sputter. Pictures of microspheres were taken by random scanning of the stub.

#### Percent recovery yield and encapsulation efficiency of microspheres

Percent recovery yield[10] of microspheres was calculated from the formula:

$$\%\;\mathrm{Yield}=\left[\frac{\mathrm{Total}\;\mathrm{weight}\;\mathrm{of}\;\mathrm{microspheres}}{\mathrm{Total}\;\mathrm{weight}\;\mathrm{of}\;\mathrm{drug},\;\mathrm{polymer}\;\mathrm{and}\;\mathrm{other}\;\mathrm{excipients}\;\mathrm{if}\;\mathrm{added}}\right]\times 100$$

Encapsulation efficiency of the microspheres was evaluated by deriving percent drug encapsulation. The drug content of drug-loaded microspheres was determined by dispersing 100 mg of microspheres in 50 ml ethanol followed by agitation with a magnetic stirrer for about 30 min to dissolve the polymer and to extract the drug. After filtration through a 5 µm membrane filter, the drug concentration in the ethanol phase was determined by taking the absorbance of this solution spectrophotometrically at 276 nm. Eudragit^®^ S100 and ethyl cellulose did not interfere under these conditions. Drug concentration was then calculated. Thus, the total drug encapsulated in total yielded microspheres from the procedure was calculated. It was expressed in percentage called as “Percent drug encapsulation” calculated as:

$$\%\;\mathrm{Drug}\;\mathrm{encapsulation}=\left[\frac{\mathrm{Actual}\;\mathrm{drug}\;\mathrm{content}}{\mathrm{Theoretical}\;\mathrm{drug}\;\mathrm{content}}\right]\times 100$$

#### *In vitro* drug release studies and comparison of release profile with marketed formulation

The drug release rate from microspheres was determined using USP XXIV basket-type dissolution apparatus.[11] A weighed amount of microspheres equivalent to 5 mg drug was filled into a capsule (size 0) and placed in the basket. Dissolution medium used was 0.1 N HCl (pH 1.2, 900 ml) for first hour and maintained at 37 ± 0.5°C at a rotation speed of 100 rpm. Prefect sink conditions prevailed during the drug release studies. 5 ml of sample was withdrawn at each 1 h interval; later this interval was extended to 2 h. Sample was then passed through a 5 µm membrane filter, and analyzed spectrophotometrically at 276 nm to determine the concentration of drug present in the dissolution medium. The initial volume of dissolution medium was maintained by adding 5 ml of fresh dissolution media after each withdrawal. The dissolution study was continued with using simulated intestinal fluid (pH 7.5 ± 1, 900 ml) for next 12 h. All experiments were conducted in triplicate.

## RESULT AND DISCUSSION

### Formation of microspheres

In the formulation of Glipizide microspheres, Eudragit^®^ S100 and controlled release polymer ethyl cellulose polymers were used, and mixture of ethanol, dichloromethane, and n-butanol was chosen as the solvent system. After introduction of drug and polymer solution in the aqueous PVA solution, an oil-in-water emulsion gets formed. Agitation provided by stirrer breaks the poured polymer solution into discrete droplets, forming an oil-in-water (O/W)-type emulsion where polymer and drug were still in their solution form in organic solvent. In the emulsion, the organic dispersed phase was drug with polymer solution and aqueous dispersion phase was PVA solution. As the stirring continued, the ethanol and n-butanol started to diffuse out from organic phase to aqueous phase, co-precipitating the drug and polymer at the interface of emulsion droplet. This co-precipitation of drug and polymer resulted into a shell around droplet. Dichloromethane remained entrapped within the shell of the droplet.

Kawashima *et al*.,[12] reported that when the diffusion rate of solvent from the organic phase emulsion droplet was too slow, microspheres coalesced together. In another study,[13] he reported that when it was too fast; the solvent may diffuse into the aqueous phase before stable emulsion droplets were formed, causing the aggregation of embryonic microspheres droplets. Here, incorporation of n-butanol in the solvent system declined the rate of diffusion of solvent into outer phase to achieve the critical diffusion rate. Appropriate rate of solvent diffusion gave desired porosity and morphology of microspheres. The alteration in this diffusion rate was due to different molecular weight of solvents. Higher the molecular weight, more time it will take to diffuse. Slower diffusion rate of n-butanol than that of ethanol provides more time for diffusion and ultimately for droplet formation. It improved the yield and decreased the losses due to aggregation of non-spherical emulsion droplets caused by rapid solvent diffusion. Apart from this, Lee *et al*.[14] had made another such effort in which ethanol was replaced by isopropanol to improve the method of microsphere preparation by controlling the diffusion rate of solvent, and the effect on the formation of microspheres was evaluated. In this study, it was also reported that yield of microspheres depended on the diffusion rate of ethanol and/or isopropanol into the aqueous phase. Kawashima *et al*.[15] documented that the stable formation of an O/W emulsion at the initial stage and the precipitation of polymer on the surface of the dispersed droplet were the key elements in formulation of microspheres with desirable morphological characteristics.

Larger amount of aqueous dispersion phase (1000 ml) was used with the intension to harden the microspheres in shorter period of time. As reported by Jain *et al*.,[10] using larger amounts of aqueous phase (400-500 ml), the diffusion of dichloromethane into the aqueous phase and hence solidification of particles occurs faster as compared to 200 ml. Thus, using large volumes of aqueous phase had potential advantage of reduction in required stirring times. Hence, diffusion of the organic solvents completed in the time span of 20 min and the microspheres get hardened.

### Micromeritic properties

Microspheres were found to be spherical and discrete. But the particle size of microspheres varied in range. The particle size increased with increase in ethyl cellulose concentration. The particle sizes of various batches of microspheres were in the range of 71µm to 474µm. Particle size range, densities, and flow properties of microspheres of batches F1-F5 are shown in Table 2.

**Table 2:** Effect of various polymer ratios over micromeritic properties of microspheres

Formulation codes	Particle size range (µm)	Bulk density* (g/ml)	Tapped density* (g/ml)	Angle of repose* (degrees)	% Compressibility*	Hausner’s ratio*
F1	142-474	0.324 ± 0.009	0.346 ± 0.009	21.52 ± 1.911	6.37 ± 1.720	1.056 ± 0.041
F2	113-457	0.339 ± 0.012	0.359 ± 0.007	21.68 ± 1.785	6.51 ± 1.913	1.061 ± 0.058
F3	92-422	0.341 ± 0.016	0.361 ± 0.011	21.18 ± 1.613	6.55 ± 1.896	1.047 ± 0.068
F4	86-326	0.348 ± 0.009	0.375 ± 0.009	22.47 ± 1.574	6.69 ± 2.045	1.072 ± 0.034
F5	71-289	0.351 ± 0.011	0.382 ± 0.006	21.55 ± 2.148	6.73 ± 1.843	1.067 ± 0.037

* Average of three preparations ± SD

Flow properties of batches were evaluated by measuring the angle of repose and compressibility index. In the evaluation of flowability of dry solid, the substance shows excellent flowability and performance, when the angle of repose have the value less than 25°, while when compressibility index has value below 9%, no aid is needed for enhancing the flowability of powder.[16] Thus, angle of repose and compressibility index are indicative of good flowability of microspheres, showing no need for addition of glidants to enhance flowability. The better flow property of microspheres indicates that the microspheres produced were non-aggregated. The improved micromeritic properties of formulated microspheres when compared to that of the pure drug alone suggest that they can be easily handled and filled into a capsule.

### Morphology

**Figure 1:**
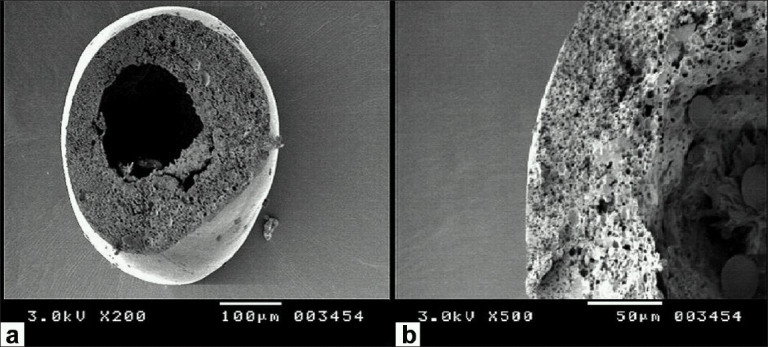
Scanning electron photomicrographs of Glipizide-loaded microspheres with cross-sectional area, (a) resolution 200 times, (b) resolution 500 times.

**Figure 2:**
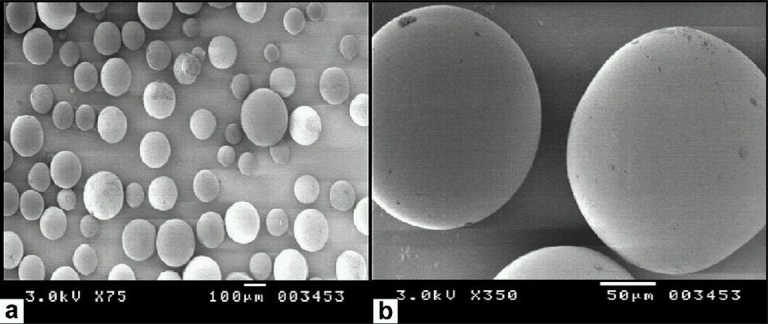
Scanning electron photomicrographs of Glipizide-loaded microspheres with surface view, (a) resolution 75 times, (b) resolution 350 times.

Surface properties and internal structure of microspheres had been revealed by scanning electron microscopy (SEM). The microphotographs of cross section and surface view of microspheres of batch F3 are shown in Figures 1 and 2.

The cross-sectional photomicrograph of the microspheres are shown in Figure 1, part (A) shows the round cavity surrounded by the thick shell of the microspheres, while part (B) shows the thick shell having about 80 µm length. Smooth outer surface of the microspheres appearing from the part (B) of the Figure 2 indicates no precipitation of drug on the surface of microspheres. SEM indicated that the microspheres produced by the emulsion solvent diffusion-evaporation method are spherical with smooth surface and not aggregated. Their smooth surface indicated that Glipizide was embedded in the shell, as the drug particles were not present on the surface.

### Percent recovery yield and encapsulation efficiency of microspheres

Percent recovery yield was found to be increased from batches F1 to F5 with an increase in concentration of Eudragit^®^ S100. It ranges from 74.81% to 96.26%, with highest recovery yield with batch F5. Percent recovery yield and percent encapsulation efficiency of the batches F1-F5 are shown in Table 3.

**Table 3:** Effect of various polymer ratios on characteristics of microspheres

Formulation batches	% Recovery yield*	% Encapsulation efficiency*	% Drug release at 12^th^ hour*
F1	74.81 ± 2.72	30.12 ± 2.23	25.19 ± 1.58
F2	86.18 ± 3.02	78.91 ± 2.34	72.18 ± 2.11
F3	89.12 ± 3.41	94.84 ± 2.41	96.76 ± 2.58
F4	94.14 ± 2.82	67.37 ± 2.06	99.54 ± 2.18
F5	96.26 ± 2.15	26.79 ± 2.57	100.91 ± 2.86

* Average of three preparations ± SD

The effect of the combination of the polymers over encapsulation efficiency was convincing. The encapsulation efficiency was found to be abruptly increasing when both polymers were used together. Encapsulation efficiencies of batches F1-F5 ranged from 26.79% to 94.84%. Maximum encapsulation efficiency was observed of the batch F3, where ratio of 2:3 of the ethyl cellulose and Eudragit^®^ S100 was used. It was about three times higher than that of batches F1 and F5 where ethyl cellulose and Eudragit^®^ S100 were used alone, respectively. This ratio of polymers was found to be the efficient of encapsulating maximum drug than any other batches.

It was reported in the literature that the encapsulation efficiency depends on the solubility of the drug in the solvent and continuous phase. An increase in the concentration of polymer in a fixed volume of organic solvent resulted in an increase in encapsulation efficiency.[17] As we have seen in the formulation, alcohol diffused out first to the external aqueous phase, thus when the drug was soluble in alcohol, it was possible that the drug may diffuse out of emulsion droplets together with alcohol before the droplet solidification, leading to a low loading efficiency. This tendency of the drug would become more prominent when the solubility of the drug in dichloromethane was low, since the drug preferentially partition into the alcohol phase when it moved into aqueous phase from a solvent mixture. In contrast to this condition, the drug Glipizide was water insoluble, along with that Glipizide was practically insoluble in alcohol and soluble in dichloromethane[18] showing greater encapsulation efficiency.

### *In vitro* drug release studies

**Figure 3:**
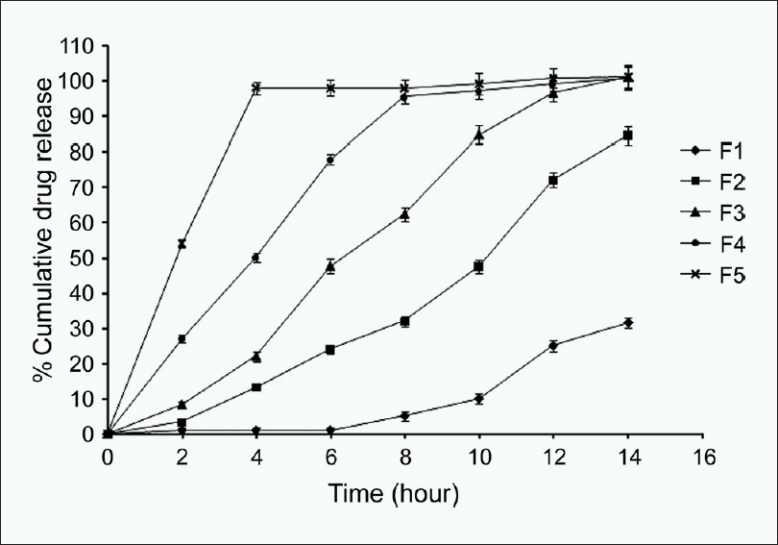
*In vitro* drug relase study of batches F1(-♦-), F2(-■-), F3(-▲-), F4(-●-), F5(-x-). Reproduction size should be column width

Different release profiles were observed with each combination of polymers. The effect of changes in polymer proportion in batches F1 to F5 has been shown in Table 3 and Figure 3. When ethyl cellulose alone was used (F1), no drug release was observed till 8th hour of dissolution study. The reason for this may be the insolubility in water and hydrophobicity of the ethyl cellulose.[19] When 1:1 proportion of ethyl cellulose: Eudragit^®^ S100 was used (F2), 70-75% drug release was observed in 12 h. This batch was controlling drug release more than 12 h. In batch F4, total drug release observed merely after 8 h. Eudragit^®^ S100 alone gave formulation (F5) which released the entire drug only in 4-5 h. One of these formulations (F3) prepared using 2:3 ratio of polymer (Ethyl cellulose: Eudragit^®^ S100) gave the most satisfactory results with extended drug release for approximately 12 h and highest encapsulation efficiency. Figure 3 had shown that increase in concentration of ethyl cellulose decreased the drug release rate. The appropriate combination of these two polymers had been achieved in batch F3 where extended release of drug for approx. 12 h had been attained.

The results of the *in vitro* drug release study obtained from batch F3 were plotted using kinetic models. Zero-order kinetics, first-order kinetics, Higuchi’s matrix, Korsmeyer Peppas model, and Hixson Crowell kinetic model were used to evaluate the release mechanism from Glipizide microspheres. The kinetic model showing highest correlation coefficient was considered as the most appropriate model for the dissolution data. The best fit with the highest correlation coefficient was observed in the Korsmeyers-peppas model and zero-order release kinetics followed by Higuchi model, as given in Table 4. The ‘*n*’ value of formulation was found to be 0.960 indicating that the drug release was followed by anomalous (non-fickian) diffusion.

**Table 4:** ‘*r*^2^’ values of various kinetic models and value of ‘*n*’

Kinetic models	Zero order	First order	Higuchi	Hixon crowell	Korsmeyers-peppas	
	*r*^2^	*r*^2^	*r*^2^	*r*^2^	*r*^2^	*n*
	0.988	0.941	0.970	0.954	0.989	0.960

## CONCLUSION

In this study, stable sustained release Glipizide microspheres were prepared successfully using the emulsion solvent diffusion-evaporation method. This study has been a satisfactory attempt to formulate a microparticulate system of an anti-diabetic drug Glipizide with a view of sustained delivery of the drug. Moreover, the developed product is less complex with regards to formulation components and processing aspects.

It may be concluded that capsules of sustained release Glipizide microspheres would be a promising drug delivery system for oral administration of Glipizide to sustain the drug release for about 12 h enhancing the patient compliance. In the formulation, the combination of cost-effective and biocompatible polymers Eudragit^®^ S100 and Ethyl cellulose had been successfully used and there is scope of scale up of the batches to the commercial level. The formulation was found to be efficient with good recovery yield and percent drug entrapment. The surface structure, particle size, and flow analysis revealed that the microspheres showed good flow and packability, indicating that it can be successfully handled and filled into a capsule dosage form.

Hence, the SR microsphere formulation of Glipizide may provide a convenient dosage form for achieving best performance regarding flow, drug entrapment, and release. Further, their potential to improve Glipizide bioavailability in humans needs to be investigated in further studies.
